# Declines and pronounced state-level variation in clozapine use among Medicare patients

**DOI:** 10.1371/journal.pone.0328495

**Published:** 2025-08-18

**Authors:** Luke R. Cavanah, Maria Y. Tian, Jessica L. Goldhirsh, Leighton Y. Huey, Brian J. Piper

**Affiliations:** 1 Geisinger College of Health Sciences, Scranton, Pennsylvania, United States of America; 2 Behavioral Health Initiative, Geisinger College of Health Sciences, Scranton, Pennsylvania, United States of America; 3 Center for Pharmacy Innovation and Outcomes, Geisinger College of Health Sciences, Danville, Pennsylvania, United States of America; Harvard University, UNITED STATES OF AMERICA

## Abstract

Schizophrenia-spectrum disorders are debilitating and contribute to a substantial economic burden. Clinicians have historically underutilized clozapine, an atypical antipsychotic traditionally reserved for treatment-resistant schizophrenia, due to the medication’s adverse effect profile and associated management requirements, concerns of complications from poor treatment adherence, and inadequate training/exposure to its use. In addition to alleviating schizophrenia symptoms when multiple other medications have failed, clozapine has other areas of demonstrated effectiveness, such as reduced suicide ideation and action, aggression, substance use, and all-cause mortality benefits that compel its use. This study aimed to characterize clozapine utilization by United States (US) Medicare patients from 2015 to 2020. Additionally, we identified the states that prescribed significantly different amounts than the national average. We observed a steady decrease in clozapine use adjusted for population (−18.0%) and spending (−24.9%) over time. For all years, there was pronounced geographic heterogeneity (average: nine-fold) in population-corrected clozapine use. Massachusetts (2015−20: 95.4, 82.7, 76.8, 72.2, 71.2, 63.7 prescriptions per thousand enrollees) and South Dakota (2015−20: 78.0, 77.4, 78.4, 75.6, 72.0, 71.6) were the only states that prescribed significantly more than average, and none prescribed significantly less. Clozapine use by US Medicare patients is low, decreasing, and concerning for underutilization—patterns previously identified for US Medicaid recipients. Further study of the reasons for the pronounced state variation is needed. Education interventions, training reform, and devices that ease required routine blood monitoring are all practical solutions to optimize clozapine use.

## Introduction

Schizophrenia-spectrum disorders are severe psychiatric disorders characterized by episodes of psychosis and functional impairment corresponding to symptom onset [1]. They are often, but not always, chronic, and may or may not feature continued decline after the functional impairment noted at symptom onset [1]. The age-standardized point prevalence and burden of schizophrenia in 2016 in the US were estimated to be 0.30% and 191.5 years lost to disability per hundred thousand, respectively [2]. About one-third of cases of schizophrenia are deemed treatment-resistant (TRS), meaning two or more antipsychotics (APs) of adequate dose, trial length, and adherence failed to resolve the symptoms [3]. Even with the low prevalence, schizophrenia results in a massive and multifaceted burden: reduced quality of life, cognitive and social functioning, and lifespan; increased depression, substance use disorder, medical comorbidities; greater need for a caregiver; and increased likelihood of experiencing unemployment and homelessness [4]. Further, schizophrenia negatively impacts the psychosocial and financial aspects of the families and caregivers [4]. Economically, the magnitude of the burden is similarly large and diverse, costing the US $155.7 billion, 24% of which is due to direct healthcare costs, 6% to direct non-healthcare costs, and 76% to indirect costs [5]. TRS is estimated to cost 3–11-fold more than schizophrenia which is treatment-responsive [6].

Clozapine is a second-generation (atypical) AP that is indicated for the treatment of TRS and the related treatment-resistant schizoaffective disorder, as well as suicidality in schizophrenia-spectrum disorders [7–10]. Not only is clozapine an effective medication when first- and second-line treatments have failed [11], it is the only indicated and evidence-based treatment for TRS [7–10,12]. Even more, a recent meta-analysis and systematic review indicates that clozapine may be more effective than other AP even when used as first- or second-line treatment for schizophrenia [13], which is an important finding since there is some speculation that TRS is a neurobiologically distinct disorder from treatment-amenable schizophrenia [14,15].

In addition to being the only established effective treatment for TRS [7–10,12] and having superior efficacy compared to other medicines in its class [13], clozapine has other areas of established efficacy. Clozapine has a strongly supported anti-suicide effect in schizophrenia/schizoaffective disorder patients, and there is even some evidence that it has a similar effect in severe and treatment-resistant suicidality and non-suicidal self-injury (NSSI) in patients with bipolar disorder or borderline personality disorder [16]. The anti-suicide benefit of clozapine is important because patients with schizophrenia, TRS, bipolar disorder, and borderline personality disorder all have significantly elevated suicide risk [17–19]. The possible NSSI benefit is important because it is a strong predictor of suicide [20], about one-fifth of people will engage in NSSI at some point in their life [21], it increases the risk of suicide [22], and is refractory to numerous psychological and pharmacological therapies [23]. Another unique but less supported benefit of clozapine is reduced aggression in patients with schizophrenia-spectrum disorders [24].

While clozapine is a highly effective medication [7–10,12] with some unique therapeutic benefits [16,24], the decision for a patient to be treated with clozapine requires a nuanced weighing of the balance of the risks of the adverse effect profile and inconveniences of blood monitoring with the possibility of recovery. Clozapine utilization remains limited by its tolerability, concerns of serious adverse effects, mandatory blood tests, perceived likelihood of patient nonadherence, clinician challenges in recognizing appropriate patients, and insufficient training and exposure to clozapine therapy [25,26]. Like other atypical APs, clozapine has the risk of metabolic syndrome, constipation (14–25%), orthostatic hypotension, sedation (≤46%), sexual dysfunction, and extrapyramidal symptoms (EPS) [27]. Of note, clozapine has a higher risk of metabolic syndrome [28] and the lowest risk of EPS [29,30] relative to other pharmaceuticals in its class. The most concerning adverse effects of clozapine, however, are leukopenia (≤3%) and agranulocytosis (1–2%) [27,31], which can lead to life-threatening infections. Cardiovascular adverse effects include dose-independent myocarditis and cardiomyopathy [27]. Neurologic adverse effects include a dose-dependent risk of seizures [27]. While it is advised that practitioners regularly monitor white blood cell and absolute neutrophil counts, between 2015 and February 2025, the FDA required enrollment in a national registry with strict monitoring requirements [7,32]. Despite clinicians’ well-founded concerns of clozapine toxicity, especially the potentially fatal effect of agranulocytosis, clozapine use is associated with decreased all-cause mortality risk [33].

Clozapine use has been historically underutilized [34–41]. Delaying clozapine treatment might not only mean prolonged symptomatology due to time spent on treatments with little efficacy, but it also can reduce the effectiveness of clozapine when it is finally tried [42]. Additionally, its rate of use appears to vary substantially from region to region within a country [35–39,41] and from country to country [34]. More specifically, there has been considerable geographic variation of clozapine use in the US Medicaid system, with the most recent study showing a 13.0-fold state-level difference in 2019, with South Dakota, Missouri, and North Dakota prescribing significantly more than the national average, and no states prescribing significantly less [35]. There is also concern that Black patients are less likely to be prescribed clozapine [31]. Though high state-level variation within the US Medicaid system has been observed in three recent epidemiologic investigations of clozapine [35,39,43] and many more by other psychotropics, such as esketamine, methadone, and buprenorphine [44–46], few reasons have been set forth to understand the reasoning behind this. Like Medicaid, pronounced state variation in the U.S. Medicare system has been noted in the prescription patterns of psychotropics, such as Z-drugs, and buprenorphine [47,48], but no recent investigations have examined this for clozapine. Given that 87% of individuals with schizophrenia are covered by Medicaid or Medicare, examining the utilization of clozapine by these patient populations can be insightful [49]. Medicare, which primarily covers those over age 65, covers almost half (46%) of patients with schizophrenia [49]. Further, the proportion of those who are 65 with schizophrenia is expected to increase with the US aging population [50]. It is also important to note that many patients with schizophrenia under age 65 qualify for Medicare because their illness makes them eligible for Social Security Disability Insurance (SSDI) for 24 months [49].

In summary, use of clozapine, the only indicated and empirically supported treatment for TRS [7–10,12], has been low and incongruent with recommendations due to the medicine’s tolerability, perceived toxicity, required blood monitoring, and lack of training in recognizing candidates for clozapine and managing patients treated with clozapine [25,26]. Considering the superior efficacy, reduced risk of suicidality and tardive dyskinesia associated with other APs, recognized effectiveness in TRS, and established history of suboptimal and geographically variable utilization of this life-saving medication, there is a need to examine the pharmacoepidemiologic patterns in clozapine use. This study aimed to analyze the Medicare data from 2015 to 2020 to characterize the national and regional variation of clozapine use.

## Methods

### Procedures

Prescription rates and spending for clozapine were obtained for 2015–2020 for Medicare Part D patients. Medicare Part D provides prescription drug coverage to help patients with Medicare offset the cost of medications [51]. Medicare covered just under 20% of the US population in 2020 [52], and about three-quarters of those with Medicare have Part D coverage [51]. Moreover, Medicare covers about 95% of non-institutionalized persons age 65 and older [53]. Prescriptions were defined as the number of Medicare Part D claims, and spending data were defined as drug costs paid for all associated claims. State-level data were extracted from Medicare Specialty Utilization and Payment Data [54]. The study was approved as exempt by the Geisinger IRB.

### Data analysis

Patterns of prescriptions, per thousand enrollees, and spending for clozapine were compared for generics, brands, and their total. States’ annual population-corrected prescriptions were then compared to the mean for that respective year, and values outside a 95% confidence interval (mean ± 1.96 * SD) were considered significantly different. The fold difference between the highest and lowest prescribing states was determined.

These methods—using z-tests to identify states with significantly different prescribing rates within a given year and measuring state variation by the fold difference between the highest and lowest prescribing states—have been used in numerous prior pharmacoepidemiologic reports [35,44–48]. Next, prescription patterns in 50 US states, per thousand enrollees, were compared for percent white population. Finally, population-corrected prescription distribution in Medicare was compared to Medicaid in 50 US states for 2019 [35] by a correlational analysis. Data were analyzed and visualized using Excel, GraphPad Prism, and Heatmapper [55].

## Results

### National prescribing

**Fig 1:**
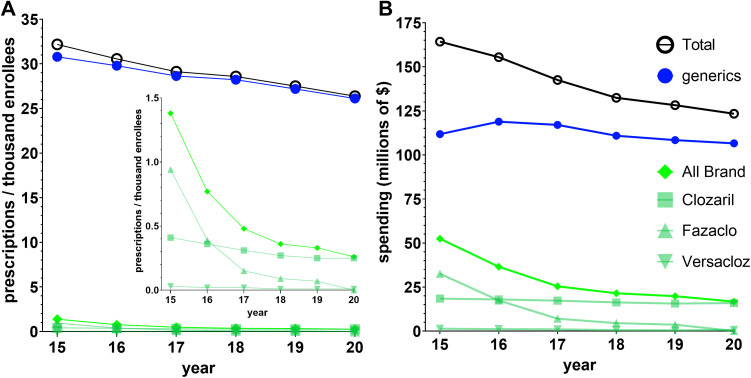
National (A) Medicare prescription rates per thousand Part D enrollees and (B) Medicare spending for clozapine gradually decreased for 2015-2020.

Fig 1A and 1B illustrate that both spending and utilization (corrected for population) gradually declined from 2015 to 2020. There were 32.2 prescriptions per thousand Medicare Part D enrollees in 2015, for which 164.3 million dollars was spent. In 2020, prescriptions per thousand enrollees were reduced by 18.0% to 26.4, while spending was reduced by 24.9% to $123.4 million. There was a decrease of 4.1% in prescriptions per thousand enrollees pre-COVID (2019) to post-COVID (2020). Moreover, Fig 1A and 1B also illustrate that the observed gradual decrease in utilization and spending applies to generics and all brand formulations. Generic clozapine accounted for the vast majority (95.7%) of prescriptions in 2015, and this increased to 99.0% in 2020 (Fig 1A). Spending on the brand was disproportionate to its use for 2015–2020. Brand name formulations comprised 31.9% of spending in 2015 and decreased to 13.6% by 2020 (Fig 1B).

### State prescribing

**Fig 2:**
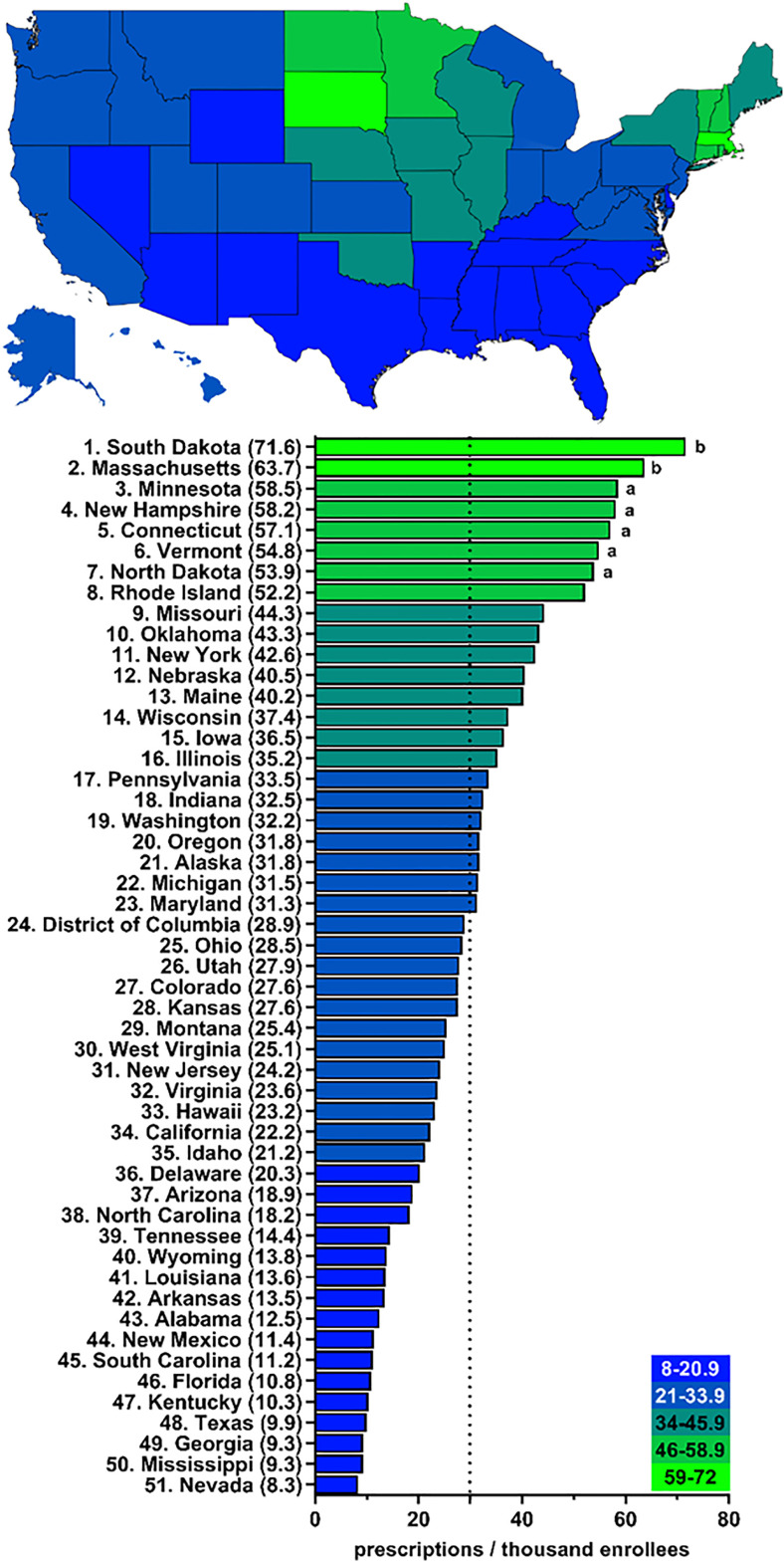
Large state-wide variation (8.6-fold) in number of clozapine prescriptions per thousand Medicare Part D enrollees in 2020 ^a^ indicates ≥1.50 SD (16.0) from the mean (29.9), which is denoted by the dotted line. ^b^ indicates ≥1.96 SD from the mean.

**Fig 3:**
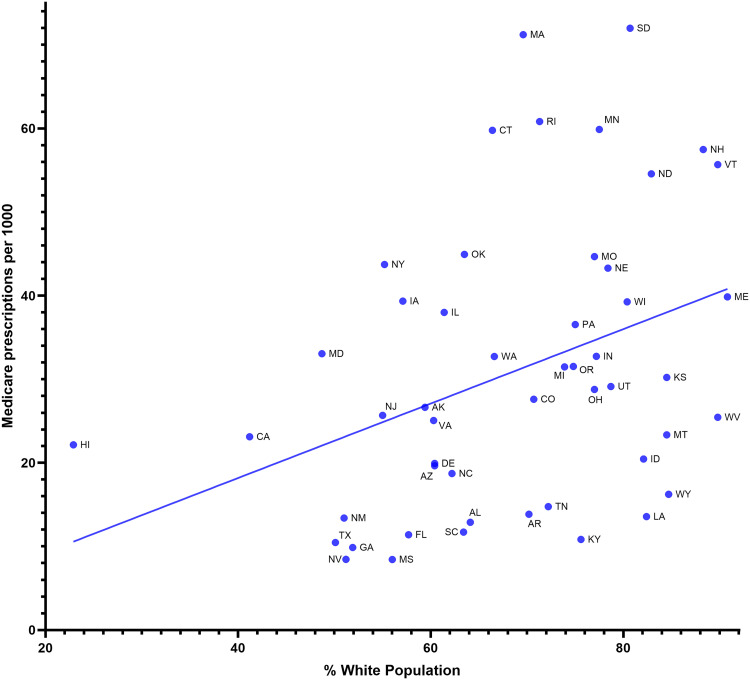
Moderate correlation (R^2^ = 0.136 p = .0085) between Medicare prescriptions per 1,000 persons and the percentage of white population among the fifty US states.

Figs 2 and S1–S5 show that, throughout the decline in spending and population-corrected use of clozapine, there existed marked variability in 2015 (11.1-fold), 2016 (10.2-fold), 2017 (8.6-fold), 2018 (8.6-fold), 2019 (8.5-fold), and 2020 (8.6-fold) from one state to another. Massachusetts was the largest prescriber in 2015 (95.4 prescriptions per thousand enrollees) and 2016 (82.7), and South Dakota was the largest prescriber in 2017 (78.4), 2018 (75.6), 2019 (72.0), and 2020 (71.6). Both Massachusetts and South Dakota prescribed significantly more than average for all years examined. Additionally, New Hampshire prescribed significantly more than average in 2015 (76.5). No state prescribed significantly less than average, although some states were below the 1.5 SD cutoff. We observed a moderate correlation (*r*(50) = 0.37, *p* = .0085, Fig 3) between the prescriptions per thousand enrollees and the percentage of white population per state. South Dakota had the highest Medicare prescriptions per thousand enrollees (72.0) while Mississippi had the lowest (8.4) (Fig 3).

**Fig 4:**
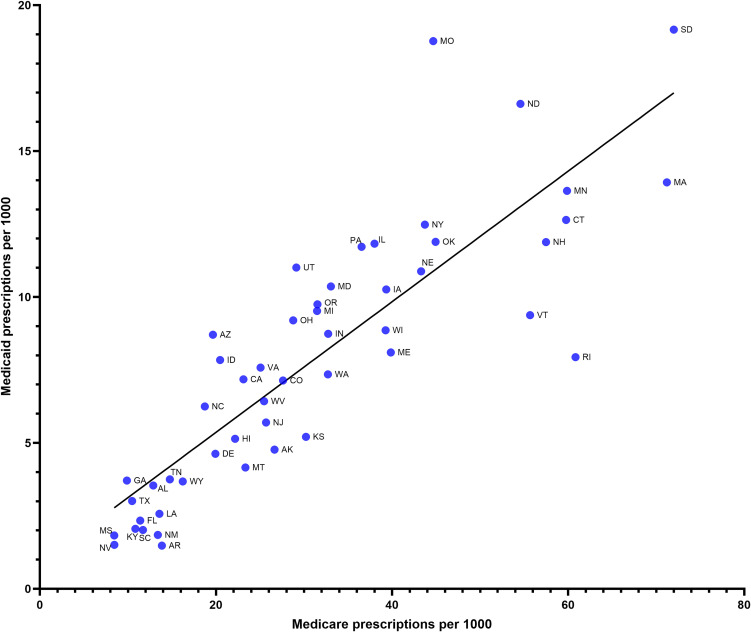
Strong correlation (R^2^ = 0.734 p < .0001) between Medicare prescriptions per 1,000 persons and Medicaid prescriptions per 1,000 persons among the fifty US states in 2019.

The prescription rates between Medicare and Medicaid correlated strongly (*r*(50) = 0.73, p < 0.0001, Fig 4). In both Medicaid and Medicare programs in 2019, South Dakota prescribed the most, exceeding the average of Medicaid state prescriptions by 59.3% and Medicare state prescriptions by 57.1% (Fig 4). In Medicaid, Arkansas had the lowest prescription of 14.8 (Fig 4). In Medicare, Mississippi had the lowest prescription of 8.4 (Fig 4).

## Discussion

There were two key findings from this novel study. First, as hypothesized, clozapine use in Medicare patients, like that of Medicaid patients [35], was low and decreasing from 2015 to 2020. The second key finding was that, as hypothesized, clozapine use in Medicare patients, like that of Medicaid patients [35], exhibited substantial geographic variability from 2015 to 2020.

### National prescribing

From 2015 to 2020, clozapine use decreased by 18% in Medicare patients. The low clozapine use is suggestive of clozapine underutilization, a finding noted in the previous studies, including the US and other nations [34–41]. The literature previously has attributed clozapine underutilization to patients’ intolerability of the adverse effects, clinician’s concerns of toxicity and patient non-adherence, requirement for patients to be placed on national registry and undergo routine blood monitoring, under-recognition of appropriate clozapine candidates, and poor training and exposure to clozapine use [25,26], which is likely the underlying explanation for these present findings as well.

Since clozapine underutilization has been a historically consistent phenomenon that has substantial consequences for patients with psychotic disorders, their families and caregivers, and the economy at large [4–6], many studies have proposed solutions to enhance the use of clozapine to better match the recommendations of clinical guidelines [56–58]. First and perhaps the most obvious solution to this problem is through education. Education with emphasis on clozapine’s comparative efficacy, anti-suicide effects, anti-aggression benefits, and lower risk of EPS is a reasonable adjustment to psychiatric training. Introducing a mandatory certification program for initiating and maintaining clozapine treatment, or requiring exposure to clozapine clinics during psychiatric residency, could significantly improve clozapine utilization [57,59]. A second possible solution for remedying clozapine under-prescription is the development of clinical tools that ease the burden of the associated medical management, especially the routine blood work. Solutions that have been tried and seem promising are tools that allow the measurement of clozapine plasma concentrations and white blood cell count through a finger-stick capillary sample, like how patients with diabetes can monitor their blood sugar [56,58]. The recent removal of the requirement for patient enrollment in a national registry with strict ANC monitoring requirements may also contribute to improved access to clozapine therapy [32].

Although these findings are concerning for clozapine underutilization by Medicare Part D enrollees, the available Medicare data does not include the indication for why clozapine was used. Future studies that differentiate epidemiologic patterns by indication of clozapine use would overcome this limitation. Additional future directions that could augment this study’s findings on chronological variation include (1) estimating how the humanistic and economic burden of TRS would be reduced if clozapine use aligned with clinical guidelines and (2) characterizing the chronological variation of other treatment strategies for TRS.

### State prescribing

Clozapine use in Medicare patients experienced an average nine-fold difference in geographic variability from 2015 to 2020. The use of clozapine also correlated strongly among the fifty US states between Medicare and Medicaid (Fig 4). Although there was a similar magnitude of geographic variation within the US Medicaid population [35], the states that prescribed significantly different amounts than average were partially distinct. While geographic disparities in clozapine use have been well documented [34–39,41], their origins are complex and not well-understood. Some of the state-level variation may be explained by differences in preferred drug lists or state policy, though further research is needed. Patient characteristics associated with clozapine initiation include: male sex, younger age, white race (Fig 3), greater outpatient service use for schizophrenia, increased mental health hospitalizations, and most significantly, residence in a county with historically high clozapine use [39].

Another possible contributor to geographic differences in clozapine utilization includes differences in the percent of non-white population, which was found to be moderately correlated (*r* = +.37, *p* < .01) with clozapine prescriptions in our study (Fig 3). Similarly, a prior report for Medicaid patients also determined that states with a higher white population prescribed more (*r* = +.38, *p* < .01) clozapine [35]. It is well-known that there are racial and ethnic disparities in the use of various medications used to treat schizophrenia [60,61], especially clozapine. The reasons for these racial and ethnic disparities are unclear, but thought to be multifactorial, including patient distrust in the medical system, clinician biases, clinician concern for benign ethnic neutropenia (BEN), and more [62]. BEN is an asymptomatic decrease in baseline neutrophil counts, which is more common among those of African descent and darker skin [63]. Importantly, the presence of BEN does not preclude clozapine treatment [64]. Improved education related to BEN and its implications on the ANC monitoring algorithm is important in preventing the underutilization of clozapine in all racial and ethnic groups.

### Future directions

Currently, concerns about clozapine’s adverse effects and the associated monitoring contribute to its underutilization. Therefore, further research into strategies for managing its adverse effects would greatly reduce the stigma around its use and help clinicians optimize the use of clozapine. The FDA’s removal of national registry and frequent ANC monitoring requirements in February 2025—previous barriers to clozapine access—warrants future study of prescribing rates before and after the policy change [32]. To develop more robust explanations of the geographical variation observed by clozapine and other psychotropic medications, it would be helpful to conduct exploratory analyses with potential causes, such as the existence of clozapine clinics, the number of psychiatrists, and other psychiatric prescribers. Further, the dataset analyzed in this study did not provide demographic information related to prescriber education or years of experience, which could provide insight into those who prescribe clozapine. Another approach to increase understanding of the geographic variation would be to investigate how the clinician’s attitudes toward clozapine (e.g., perceived toxicity, concerns of patient non-adherence) vary from one state to the next and conduct correlational analyses with state use. Qualitative studies of patients’ perspectives might also be valuable. As also noted above, since the indication of clozapine is not included in the present study’s dataset, it would be valuable to stratify the number of state population-corrected prescriptions by clozapine indication. Epidemiologic investigation of the use of other effective and underutilized psychiatric treatments and comparison of patterns to the present study’s findings may also reveal insights for possible reasons for underuse.

## Conclusion

In summary, rates of clozapine prescriptions to Medicare patients have decreased over the past six years, despite prior evidence of it having already been underutilized. Throughout this chronological decrease in prescriptions of clozapine, there was evidence of large (nine-fold) state-level variation. States with a greater make-up of non-white residents prescribed less clozapine. Future studies should explore possible associations underlying the decreasing and variable use of clozapine among Medicare patients.

## Supporting information

S1 Fig Large state-wide variation (11.1-fold) in number of clozapine prescriptions per thousand Medicare Part D enrollees in 2015 ^a^ indicates ≥1.50 SD (20.2) from the mean (36.4), which is denoted by the dotted line. ^b^ indicates ≥1.96 SD from the mean. (TIF)

S2 Fig Large state-wide variation (10.2-fold) in number of clozapine prescriptions per thousand Medicare Part D enrollees in 2016 ^a^ indicates ≥1.50 SD (19.2) from the mean (34.8), which is denoted by the dotted line. ^b^ indicates ≥1.96 SD from the mean. (TIF)

S3 Fig Large state-wide variation (8.6-fold) in number of clozapine prescriptions per thousand Medicare Part D enrollees in 2017 ^a^ indicates ≥1.50 SD (18.0) from the mean (33.1), which is denoted by the dotted line. ^b^ indicates ≥1.96 SD from the mean. (TIF)

S4 Fig Large state-wide variation (8.6-fold) in number of clozapine prescriptions per thousand Medicare Part D enrollees in 2018 ^a^ indicates ≥1.50 SD (17.9) from the mean (32.9), which is denoted by the dotted line. ^b^ indicates ≥1.96 SD from the mean. (TIF)

S5 Fig Large state-wide variation (8.5-fold) in number of clozapine prescriptions per thousand Medicare Part D enrollees in 2019 ^a^ indicates ≥1.50 SD (16.8) from the mean (31.0), which is denoted by the dotted line. ^b^ indicates ≥1.96 SD from the mean. (TIF)

S1 Data Medicare clozapine data analysis. (XLSX)
