# Supplementary figures and images for: Declines and pronounced state-level variation in clozapine use among Medicare patients
Source: PLoS One. 2025 Aug 18;20(8):e0328495. doi: 10.1371/journal.pone.0328495 (PMC12360599; doi:10.1371/journal.pone.0328495)

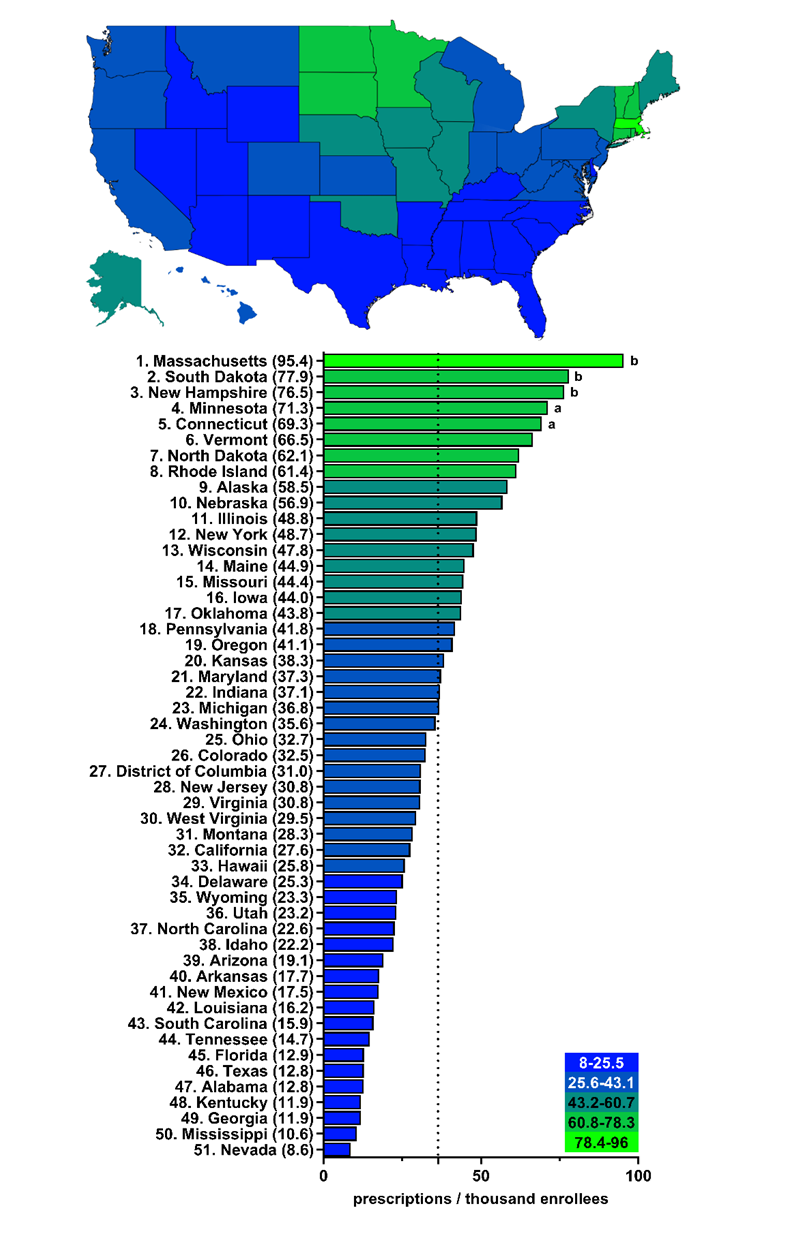

Supplement: S1 Fig — (TIF) [file pone.0328495.s001.tif]

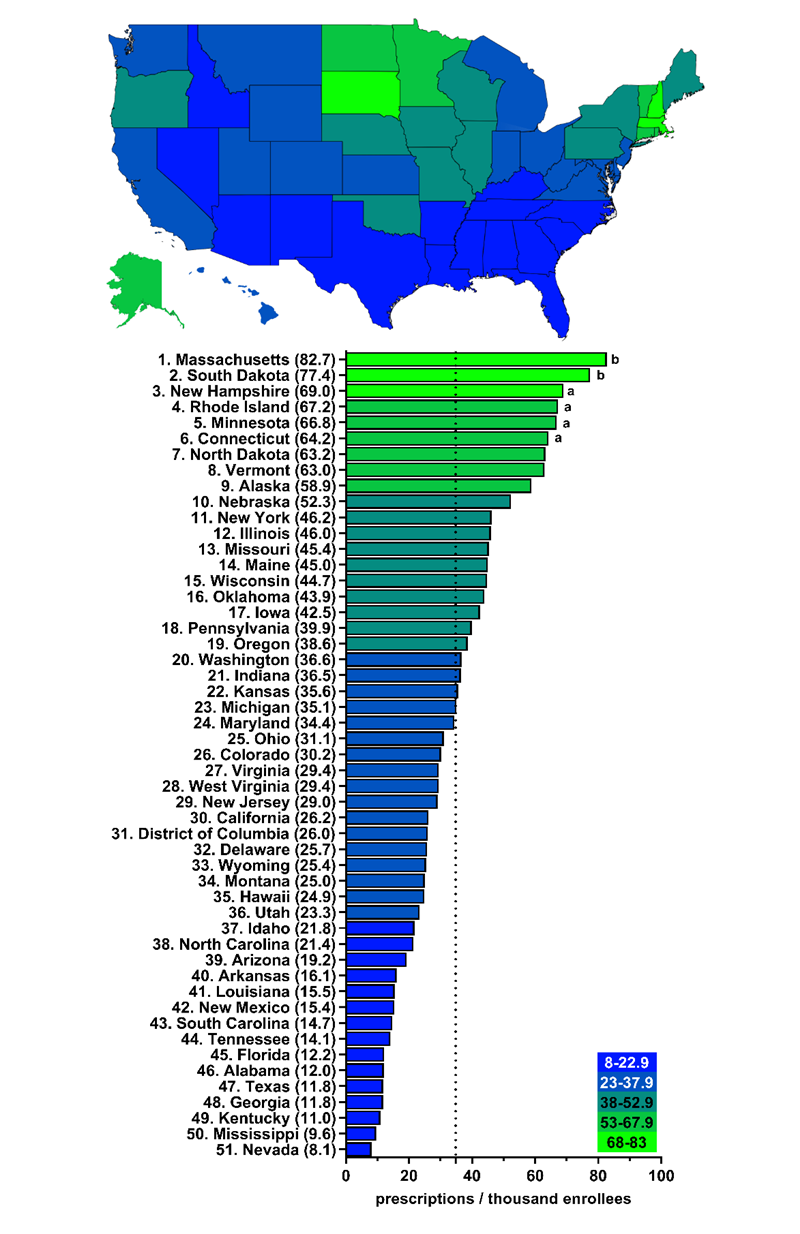

Supplement: S2 Fig — (TIF) [file pone.0328495.s002.tif]

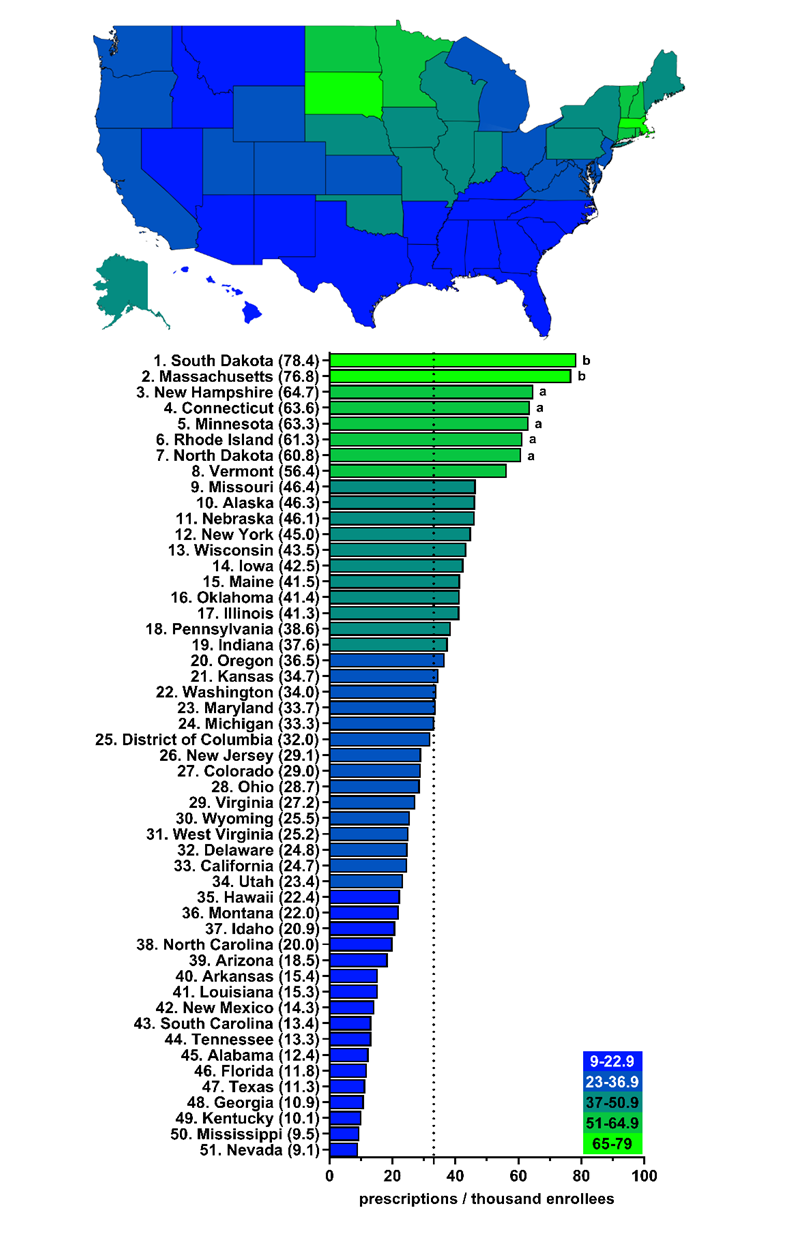

Supplement: S3 Fig — (TIF) [file pone.0328495.s003.tif]

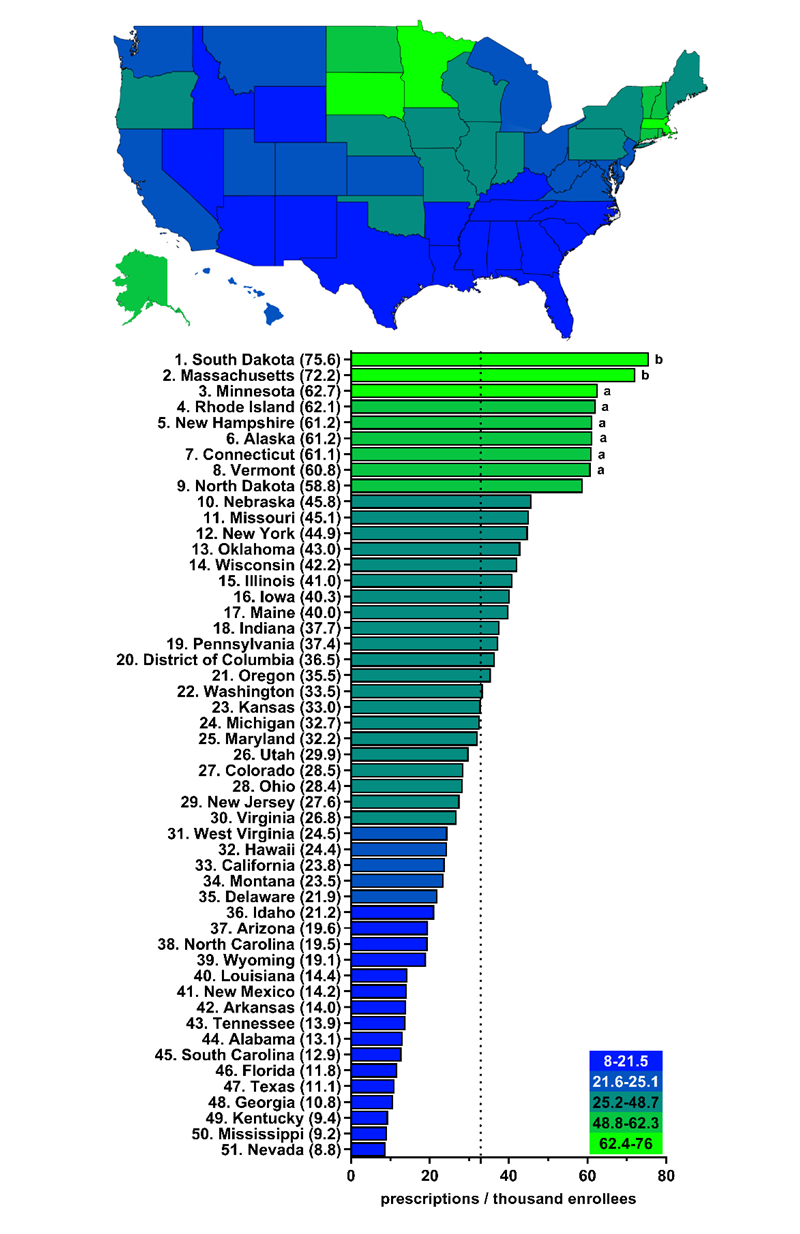

Supplement: S4 Fig — (TIF) [file pone.0328495.s004.tif]

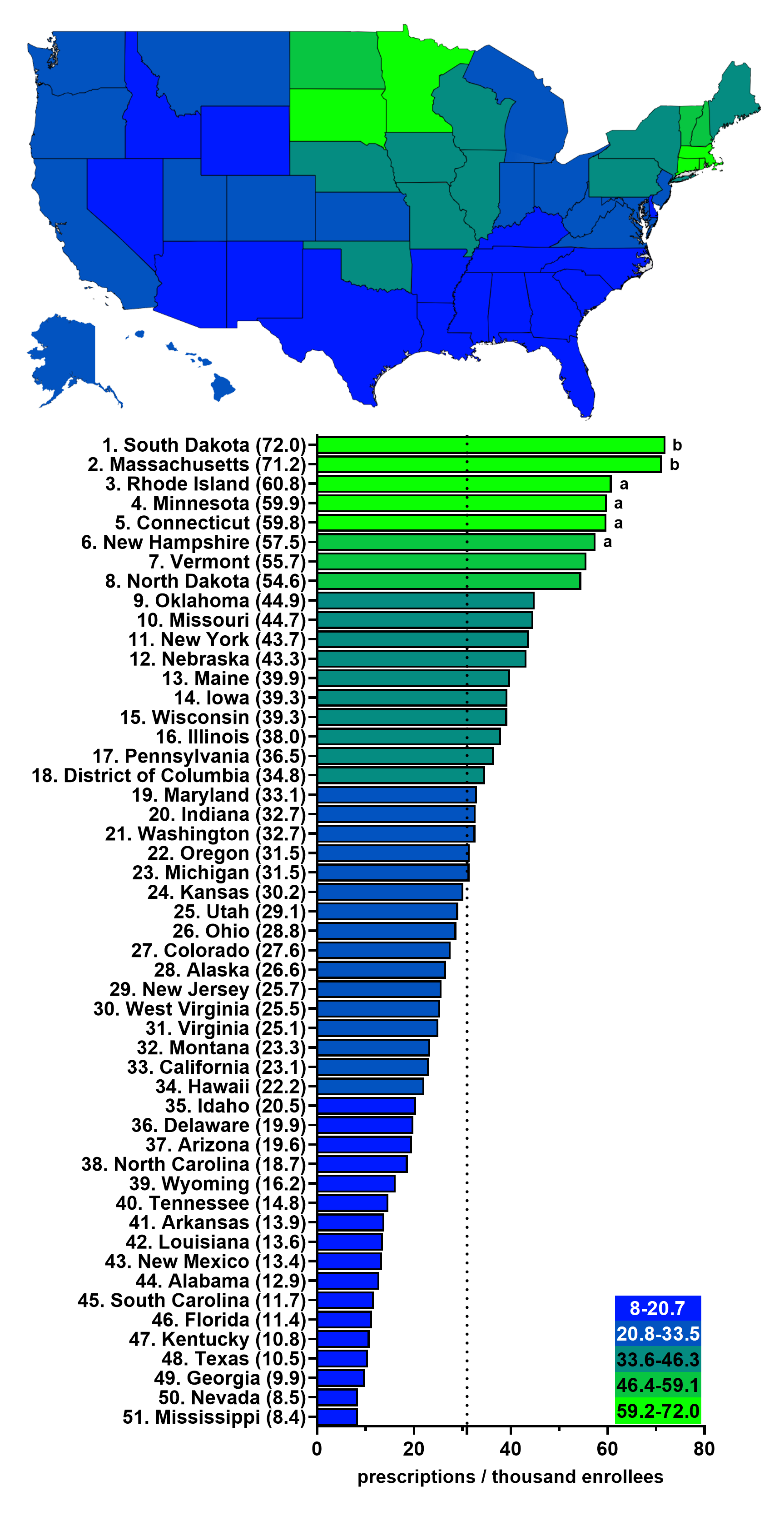

Supplement: S5 Fig — (TIF) [file pone.0328495.s005.tif]
